# From hallways to headlines: The hidden surge in youth nicotine pouch use

**DOI:** 10.18332/tpc/224299

**Published:** 2026-09-22

**Authors:** Melinda Ickes, Melissa A. Little

**Affiliations:** 1 Department of Kinesiology and Health PromotionUniversity of KentuckyLexingtonUnited States; 2 University of Virginia, School of MedicineCharlottesvilleUnited States

**Keywords:** tobacco prevention, oral nicotine pouches, youth nicotine use

**Dear Editor**,

Oral nicotine pouches (ONPs) represent one of the fastest-growing segments of the nicotine market^1^. These products are discreet, odorless and do not require spitting, characteristics that facilitate undetected use in school. Although use remains lower than e-cigarettes, the patterns of use and sales trends suggest this may represent the early stages of a significant increase in youth nicotine exposure rather than a transient phenomenon^2^.

According to the 2024 US National Youth Tobacco Survey (NYTS), 1.8% of middle and high school students (approximately 480000 youth), reported current ONP use^3-5^. Among 10th- and 12th-grade students, ever use increased from 3.0% in 2023 to 5.4% in 2024, an 80% relative increase^3^.

Several characteristics of ONPs warrant concern from a public health standpoint. Nicotine exposure during adolescence adversely affects neurodevelopment, with documented effects on attention, learning, impulse control, and mood regulation^6^. Some ONPs contain high levels of nicotine, and product design features increase the proportion of free nicotine and enhance the rate of absorption^7,8^. Flavors and nicotine formulations may increase nicotine delivery, dependence risk, and abuse potential^7-9^. Youth-targeted marketing, extensive flavor proliferation, and amplification through social media and influencer-driven content collectively increase the appeal and normalization of ONPs among youth^10-12^.

Emerging real-world classroom-level data from Kentucky, a state with historically higher tobacco use, highlight how quickly ONPs have entered the youth environment. In 2024–2025, among a convenience sample of sixth-12th graders who received the #iCANendthetrend (ICETT) near-peer tobacco prevention program (N=3167)^13,14^, 6% of high school and nearly 3% of middle school students reported ONP use the prior year; no students reported ONP use in the prior program year^15^. Additionally, youth in more rural communities reported higher ever use of ONPs^15^, suggesting emerging social-environmental disparities from distinct exposure pathways, access points, and social norms surrounding discreet nicotine products. In response, the ICETT program was adapted in 2024 to integrate explicit ONP-focused education addressing nicotine-related harms, misconceptions associated with ‘tobacco-free’ labeling, youth-targeted marketing, and addiction potential.

Similarly, in a randomized controlled trial of UP2U*Teacher,* a classroom-based brief tobacco and nicotine intervention, among 548 ninth-grade students from 10 Virginia high schools^16^, 5.4% reported ONP use. Following the intervention, perceptions of ONP harm and addictiveness increased significantly, suggesting students are receptive to this information^16^.

These data carry several implications for public health research and practice.

First, prevention curricula should explicitly address ONPs, including nicotine-related harms, marketing tactics, and the misconceptions surrounding ‘tobacco-free’ labeling. Youth perspectives should inform educational materials and peer-to-peer messaging.

Second, educators, parents, and healthcare providers need greater awareness of ONPs through school-based training, family communication resources, and routine clinical screening.

Third, longitudinal studies examining youth susceptibility, behavioral trajectories, health effects, and risk for product escalation are essential to inform regulatory action and evidence-based prevention.

Surveillance data, program observations, and accelerating sales collectively suggest a narrowing window for intervention. Coordinated efforts to modernize prevention programming, expand stakeholder engagement, and invest in targeted research will be critical to mitigating the public health consequences of this emerging product.
